# Strategies in the Management of Pancreatic Ductal Adenocarcinoma Involving Aberrant Right Hepatic Artery Arising From the Superior Mesenteric Artery

**DOI:** 10.7759/cureus.30781

**Published:** 2022-10-27

**Authors:** Dhiresh K Maharjan, Roshan Ghimire, Yugal Limbu, Sujan Regmee, Rabin Pahari, Suman K Shrestha, Prabin Bikram Thapa

**Affiliations:** 1 Department of Gastrointestinal and General Surgery, Kathmandu Medical College and Teaching Hospital, Kathmandu, NPL

**Keywords:** whipple’s operation, neoadjuvant therapy, pancreatic ductal adenocarcinoma, borderline resectable, aberrant right hepatic artery

## Abstract

Introduction

The prevailing guidelines do not include the involvement of an aberrant right hepatic artery (aRHA) arising from the superior mesenteric artery in classifying borderline resectable pancreatic ductal adenocarcinoma (BR PDAC). Our novel classification aims to distinguish different entities depending on the location and degree of tumor involvement of aRHA and propose a strategy to manage tumor involvement of aRHA in PDAC.

Material and methods

The patients who underwent pancreaticoduodenectomy (PD) from September 1, 2018, to August 31, 2022 were analyzed retrospectively, and patients with aRHA were included in the study. Depending on the radiological data, arterial involvement of the aRHA was classified into group I with proximal involvement of the aRHA up to 2 cm from its origin in the superior mesenteric artery (SMA) and group II with distal involvement of aRHA beyond 2 cm from its origin in SMA. In addition, the resection margin status was correlated with the technique employed for managing the tumor-involved artery.

Results

A total of 122 patients underwent PD during the study period. Eight patients were identified to have tumor involvement of the aRHA arising from the SMA. Among the five patients in group I, three patients who had upfront surgery showed R1 resection regardless of periarterial divestment or resection/reconstruction of the involved artery, whereas R0 resection was achieved in the two patients who had neoadjuvant therapy. All patients in group II had R0 resection regardless of receiving neoadjuvant therapy. There were no significant morbidity and mortality in our series.

Conclusion

The aRHA should be considered in the classification of BR PDAC. Management strategies should be tailored based on the location and the degree of tumor involvement in the aRHA. We advocate neoadjuvant therapy for proximal involvement and upfront surgery for distal involvement of aRHA to achieve good oncological clearance.

## Introduction

According to the International Study Group of Pancreatic Surgery (ISGPS) guidelines [1], borderline resectable pancreatic ductal adenocarcinoma (BR PDAC) with relation to arterial involvement has been described as tumor abutment of the superior mesenteric artery (SMA) not exceeding ≤180° of the circumference of the vessel wall or direct abutment of the hepatic artery (HA) without extension to the celiac axis.

A similar criterion was put forth in 2009 [2] by the American Hepato-Pancreato-Biliary Association (AHPBA), Society of Surgical Oncology (SSO), and the Society of Surgery of the Alimentary Tract (SSAT) in their AHPBA/SSO/SSAT 2009 joint consensus. However, none of these guidelines have considered the circumstance where there could be an abutment or encasement of the aberrant right hepatic artery (aRHA) arising from SMA. In this study, we aim to retrospectively classify the cohort of patients undergoing pancreaticoduodenectomy (PD) who have aRHA involvement based on their anatomical location and the degree of radiological involvement, and deduce their outcomes in terms of curative resection.

## Materials and methods

Following ethical clearance from the institutional review committee, a retrospective analysis of patients undergoing PD from September 1, 2018 to August 31, 2022 was conducted in the Department of General and Gastrointestinal Surgery, Kathmandu Medical College and Teaching Hospital, Kathmandu, Nepal. All patients were subjected to staging laparoscopy with near-infrared fluorescence imaging using indocyanine green dye to rule out peritoneal deposits and metastatic lesions prior to carrying out the resection.

Cattell-Braasch mobilization followed by an artery-first approach was carried out in all the cases. A combined arterial approach was performed by isolating the SMA and the accessory right hepatic artery, followed by early ligation of the inferior pancreaticoduodenal artery (IPDA) to minimize blood loss. After portal dissection and gastric and jejunal transection, the pancreas was divided using a bipolar cautery device, and the head and uncinate process of the pancreas were removed en-bloc with the 'C' loop of the duodenum and the proximal jejunum. Venous resection and reconstruction, when necessary, were performed by primary repair or reconstruction in an end-to-end fashion. Arterial involvement was managed by either periarterial divestment or resection and reconstruction of the involved artery. A triangle operation was performed in all cases [3]. The pancreaticojejunostomy and the hepaticojejunostomy were performed using Blumgart's technique. The gastrojejunostomy was performed in an end-to-side fashion in two layers. Based on the radiological data, arterial involvement of the aRHA was classified into two groups, as shown in Table 1.

**Table 1:** Classification based on tumor location. aRHA: Aberrant right hepatic artery; SMA: Superior mesenteric artery.

Group I – Proximal involvement of aRHA up to 2 cm from its origin in SMA	Group II – Distal involvement of aRHA beyond 2 cm from its origin in SMA
IA: Abutment only	IIA: Abutment only
IB: Encasement without luminal narrowing	IIB: Encasement without luminal narrowing
IC: Encasement with luminal narrowing	IIC: Encasement with luminal narrowing

## Results

**Figure 1:**
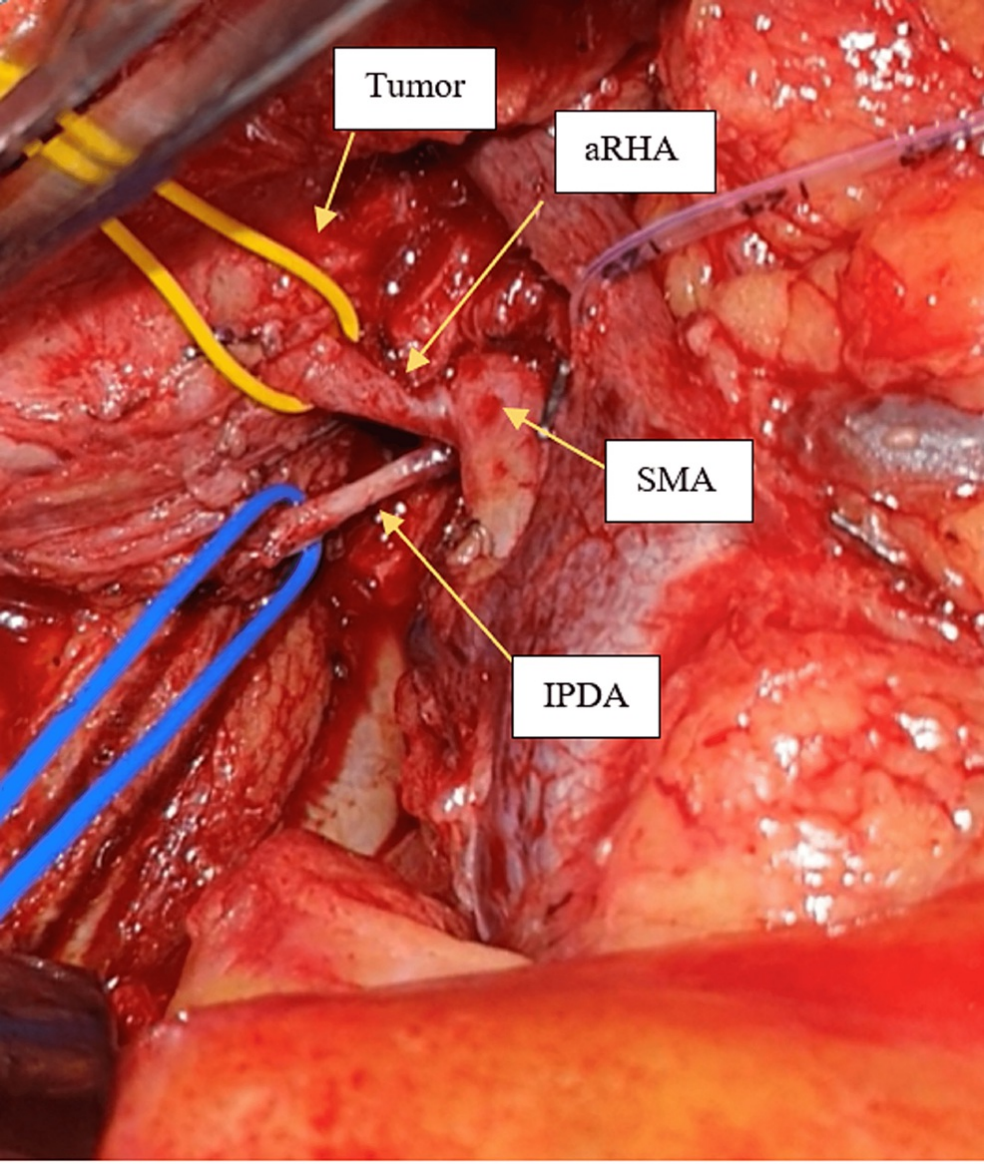
Tumor involving proximal aRHA within 2c m from its origin from SMA. aRHA: Aberrant right hepatic artery; SMA: Superior mesenteric artery; SMV: Superior mesenteric vein; IPDA: Inferior pancreaticoduodenal artery.

**Figure 2:**
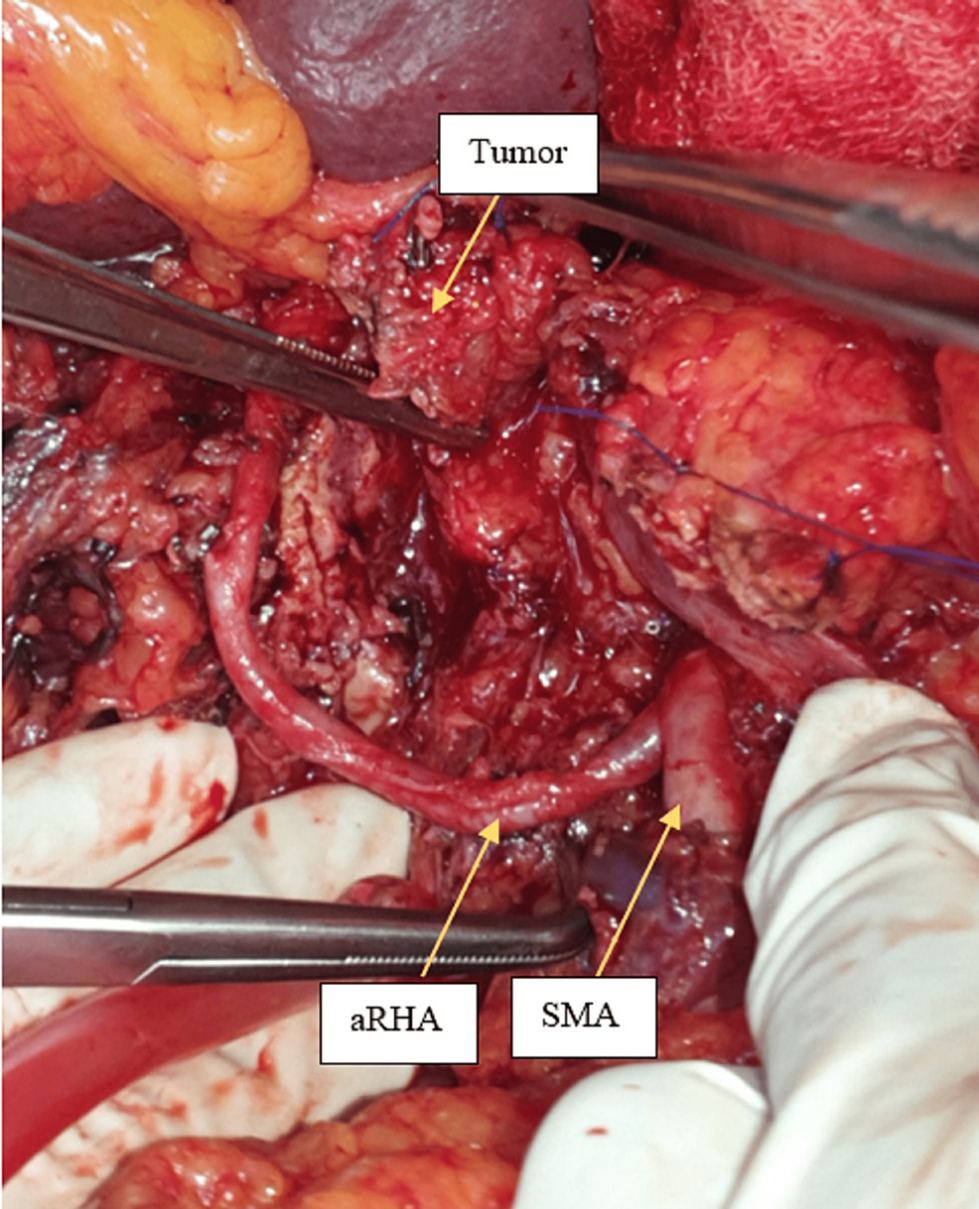
Tumor involving distal aRHA more than 2 cm away from its origin from SMA. aRHA: Aberrant right hepatic artery; SMA: Superior mesenteric artery.

Among 122 patients who underwent PD for various pathologies from September 1, 2018 to August 31, 2022, eight patients were identified to have aRHA involvement. The mean age of the eight patients was 66.6 years, and five were male. Three patients had received neo-adjuvant chemo-radiotherapy with the FOLFIRINOX regimen, and the rest underwent upfront surgery. Among five patients with proximal involvement of aRHA (group I), three underwent peri-arterial divestment, and two underwent resection and reconstruction of the artery (Table 2). Among three patients with distal involvement of aRHA (group II), two underwent peri-arterial divestment, and one underwent resection and reconstruction of the artery. In group I, all patients who underwent upfront surgery resulted in an R1 resection margin (distance of the tumor from the resection margin of < or = 1 mm) regardless of peri-arterial divestment or resection/reconstruction of the artery. However, in group II, all three patients had R0 resection (no residual microscopic tumor) despite two out of the three patients not receiving neoadjuvant therapy. The average intraoperative blood loss was 300 ml, and there were no clinically significant post-operative pancreatic fistulas in any of the patients. Among the eight patients, three had Clavien Dindo grade 1 complications, and five had grade 2 complications requiring blood transfusion. Intra-operative pictures of group I and group II tumors are shown in Figures 1-2, respectively. 

**Table 2:** Patient characteristics, management, and outcome. Ca: Carcinoma; FOLFIRINOX: Folinic acid, Fluorouracil, Irinotecan Hydrochloride, Oxaliplatin; RT: Radiotherapy, R0: No microscopic tumor remnant; R1: Microscopic resection margin positive.

Category	Age	Sex	Diagnosis	Involvement	Treatment	Arterial Procedure	Resection status
Group I	45	F	Ca head of pancreas	A: Abutment only.	Upfront Surgery	Peri-arterial divestment	R1
	67	F	Ca uncinate process of pancreas	B: Encasement without luminal narrowing.	Upfront Surgery	Peri-arterial divestment	R1
	62	M			Upfront Surgery	Resection and anastomosis	R1
	78	M			Neoadjuvant FOLFIRINOX with RT	Peri-arterial divestment	R0
	69	M	Ca uncinate process of pancreas	C: Encasement with luminal narrowing.	Neoadjuvant FOLFIRINOX with RT	Resection and anastomosis	R0
Group II	75	M	Ca head of pancreas	A: Abutment only.	Upfront Surgery	Periarterial divestment	R0
	59	F	Ca head of pancreas	B: Encasement without luminal narrowing.	Upfront Surgery	Periarterial divestment	R0
	78	M	Ca head of pancreas	C: Encasement with luminal narrowing.	Neoadjuvant FOLFIRINOX with RT	Resection and anastomosis	R0

## Discussion

Out of the eight patients with aRHA who underwent PD, five underwent peri-arterial divestment, and three underwent resection and reconstruction of the involved artery. Peri-arterial divestment was undertaken when there was arterial encasement without luminal narrowing. At the same time, arterial resection and reconstruction were opted for when there was arterial encasement with luminal narrowing. The analysis of our retrospective cohort shows that the occurrence of an aRHA is not uncommon. This finding concurs with the observation from our previous study and various other studies in which the reported prevalence rate of aRHA is 15-20% among patients undergoing various hepato-pancreatico-biliary surgeries [4,5]. The aRHA can be completely replaced or an accessory artery arising from SMA or as a direct branch from the aorta or the gastroduodenal artery [6]. We classified the location of tumor involvement as group I with the proximal first 2 cm of aRHA from its origin in SMA and group 2 involving the distal part beyond 2 cm from its origin in SMA. The results of this study suggest that this classification based on the proximal or distal location of the involvement by the tumor can be a factor in achieving R0 resection during PD. A replaced RHA from SMA should always be preserved during PD if not involved by the tumor, as its unintentional division can result in the ischemic breakdown of the hepaticojejunostomy resulting in post-operative biliary fistula [7]. However, when the aRHA arising from SMA is involved by the tumor, its preservation may compromise the oncological clearance of the tumor from SMA, leading to a high chance of R1 resection.

Although there are many studies describing the clinical outcome of arterial involvement/infiltration of SMA or CHA in borderline resectable and locally advanced pancreatic cancer, there are only a handful of studies describing the significance of the involvement of the aRHA in BR PDAC [8-11]. A study by Kim PT et al. shows that the resectability in patients undergoing PD is not affected by the presence of aRHA. In instances with isolated involvement of an aRHA, the artery was sacrificed, and when a replaced RHA was involved, it was resected and reconstructed to achieve complete resection margins [12]. Intraoperative clamping of the aRHA can be performed to assess whether the artery can be sacrificed during resection [8]. If the distal arterial supply is intact after occluding the involved artery with an atraumatic vascular clamp, the involved artery can be resected en-bloc with the specimen. However, if the clamp test shows that the distal arterial supply is entirely dependent on the involved artery, peri-arterial divestment or resection/reconstruction should be undertaken based on the degree of tumor infiltration in the artery. There have also been reports on successful resection of tumor-encased aRHA following a pre-operative coil embolization to enlarge the collateral pathways and prevent ischemia-related complications [10].

**Figure 3:**
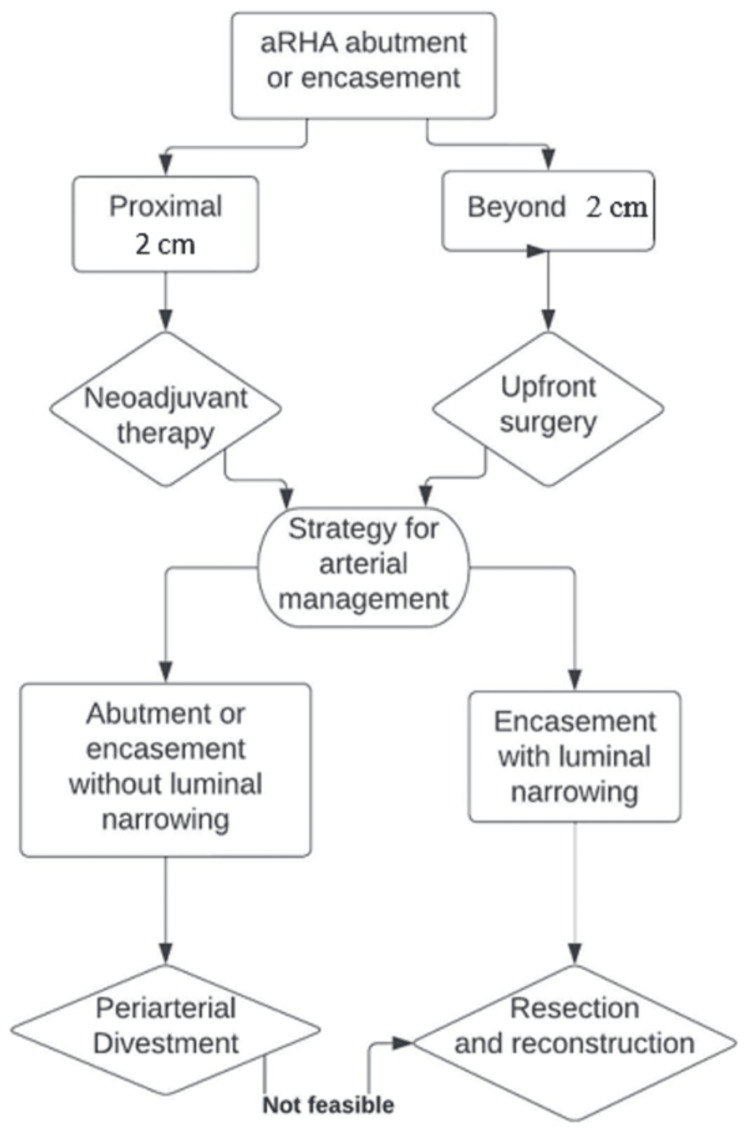
Strategy for managing aRHA involvement during PD. aRHA: Aberrant right hepatic artery; PD: Pancreaticoduodenectomy.

Peri-arterial divestment in pancreatic cancer surgery has brought a paradigm shift in the management of arteries involved by the tumor. Divestment of the involved artery increases the chance of complete tumor resection and avoids the morbidity related to vascular reconstruction in selected patients [13-15]. The same principle of peri-arterial divestment was applied in our study to manage the tumor-involved aRHA, which successfully achieved R0 resection, especially in patients with distal arterial involvement. Based on our results, our proposed hypothesis is that peri-arterial divestment would suffice to clear the resection margin when the distal portion of the aRHA is involved by the tumor. In contrast, a resection/reconstruction of the artery is warranted to achieve an R0 resection when the proximal part of the aRHA is involved (Figure 3). This knowledge can aid surgeons in making the pre-operative decision on the management plan for the arterial involvement of the tumor in BR PDAC with aRHA.

Furthermore, this study also emphasizes the need to include aRHA in the future classification of the BR PDAC by ISGPS. The chance of achieving R0 resection was better when the patient had neo-adjuvant chemo-radiotherapy, especially when the proximal part of the aRHA was involved, underscoring the importance of neoadjuvant chemo-radiotherapy in BR PDAC to achieve R0 resection margin. Of note, none of the patients had significant morbidity in our series, and none developed pseudoaneurysm, despite peri-arterial divestment and arterial resection and reconstruction. The absence of pseudoaneurysm is likely attributed to the position of the aRHA as, unlike the SMA and common hepatic artery reconstruction where the pancreatic-enteric anastomosis is in close vicinity of the arterial reconstruction, the pancreatic-enteric anastomosis is away and to the left of aRHA reconstruction. The data on the long-term survival outcomes of this series is awaited.

Our study's limitations include its retrospective nature, small sample size, and the lack of comparison to patients with sacrificed or reconstructed aRHA.

## Conclusions

The presence of an aRHA is not uncommon in patients undergoing PD and has clinical significance in managing PDAC. Peri-arterial divestment and arterial resection/reconstruction can be utilized to manage arterial involvement by the tumor. When the proximal 2 cm of the aRHA is involved, neo-adjuvant therapy increases the chance of R0 resection. In contrast, when the distal aRHA is involved, oncological clearance can be achieved with up-front surgery.
